# SARS-CoV-2 Vaccination Status and Post-Infection Cardiopulmonary Exercise Performance in Elite Male Team-Sport Athletes

**DOI:** 10.3390/jfmk11030305

**Published:** 2026-08-03

**Authors:** Srdjan Markovic, Tamara Stojmenovic, Dragan Atanasov, Dragutin Stojmenovic, Milos Markovic, Marina Ostojic, Ivana Nedeljkovic

**Affiliations:** 1 Faculty of Physical Education and Sports Management, Singidunum University, 11000 Belgrade, Serbia; tstojmenovic@singidunum.ac.rs (T.S.); datanasov@singidunum.ac.rs (D.A.); 2 Sports Medicine Clinic Vernermed, 11000 Belgrade, Serbia; dragutin.stojmenovic@gmail.com; 3 Faculty of Sport and Physical Education, University of Belgrade, 11000 Belgrade, Serbia; milos.markovic@fsfv.bg.ac.rs; 4 Faculty of Medicine, University of Belgrade, 11000 Belgrade, Serbia; drmarinaostojic@gmail.com (M.O.); ivana.nedeljkovic@med.bg.ac.rs (I.N.)

**Keywords:** COVID-19, CPET, VO_2_max, ventilatory threshold, oxygen pulse, return to play, soccer, basketball

## Abstract

**Background**: SARS-CoV-2 infection may impair functional capacity in athletes, while prior vaccination status may be associated with post-infection functional status. However, evidence comparing objective post-infection exercise responses according to vaccination status in elite athletes remains limited. **Methods**: This study included a total of 220 male athletes with PCR-confirmed SARS-CoV-2 infections: 101 unvaccinated and 119 vaccinated with two doses prior to infection. Cardiopulmonary exercise testing (CPET) was performed 14 ± 1 days after confirmation of SARS-CoV-2 infection and before the athletes returned to full training. A two-way ANOVA was conducted for each CPET variable, using vaccination status and sport as fixed factors. **Results**: After accounting for the type of sport, vaccinated athletes showed significantly higher VO_2_max, VO_2_ at VT1, oxygen pulse, heart rate at VT1, and heart rate at VT2, as well as lower VE/VCO_2_ values. No significant differences were found for VEmax, HRmax, or heart rate during recovery. The interaction between vaccination status and sport was not significant for the main CPET outcomes, indicating a similar pattern in both sports. **Conclusions**: These findings suggest that prior vaccination is associated with a more favorable early post-infection physiological profile in professional team-sport players.

## 1. Introduction

Elite athletes are particularly vulnerable to the effects of infectious diseases due to the high physical demands of training and competition [1]. Intensive training, frequent travel, and close interpersonal contact can increase exposure to pathogens, while periods of insufficient rest may transiently suppress immune function, leaving athletes at greater risk of illness and prolonged recovery periods [1,2,3]. The global SARS-CoV-2 pandemic had a profound impact on elite sports, with widespread infections causing interruptions in training, competition cancellations, and concerns regarding athletes’ functional capacity after recovery [4,5,6].

Vaccination emerged as the primary preventive strategy to reduce the incidence and severity of coronavirus disease 2019 (COVID-19) and has played a pivotal role in public health responses worldwide. In elite sport settings, vaccination has been encouraged to protect athletes’ health and maintain training availability [7,8], yet initial hesitancy and concerns about vaccine-related side effects led to variable uptake among athletic populations [9]. Although SARS-CoV-2 vaccines have been shown to have favorable safety profiles and to reduce the risk of severe clinical outcomes in the general population [8,10,11,12], questions remain regarding their impact on athletes’ functional performance, particularly following recovery from COVID-19.

Several studies have examined vaccine tolerability and short-term effects on athletic training schedules, with many athletes reporting only mild, transient symptoms and minimal training disruption after vaccination [8,10,13]. However, the consequences of SARS-CoV-2 infection for physical performance and the potential modifying influence of prior vaccination have been less thoroughly investigated in elite athletic populations. Some evidence suggests that infection may impair cardiopulmonary function and aerobic capacity, even in young and otherwise healthy individuals [4,14,15,16]. Conversely, vaccine-induced immunity may attenuate disease severity and contribute to more favorable functional recovery after infection [8,9,10,12,13]. Nevertheless, evidence directly comparing post-infection cardiopulmonary exercise performance between vaccinated and unvaccinated elite athletes remains limited.

Understanding the relationship between vaccination status and post-infection functional recovery has important implications for athletes, coaches, and sports medicine professionals. Elite athletes depend on optimal aerobic and anaerobic capabilities for performance, and even small decrements in physiological function can meaningfully affect competitive outcomes [14,17]. Cardiopulmonary exercise testing provides an objective assessment of integrated pulmonary, cardiovascular, and metabolic responses to exercise and may therefore be particularly relevant for return-to-play evaluation in professional athletes [4,5,18].

Accordingly, the primary aim of this study was to compare post-infection cardiopulmonary functional performance between vaccinated and unvaccinated elite athletes following confirmed SARS-CoV-2 infection. We hypothesized that athletes vaccinated prior to infection would demonstrate more favorable post-infection cardiopulmonary exercise test parameters than unvaccinated athletes.

## 2. Materials and Methods

This retrospective observational cohort study investigated post-infection cardiopulmonary functional performance using routinely collected medical and return-to-play data from elite athletes with confirmed SARS-CoV-2 infection. Athletes were grouped according to vaccination status at the time of infection and compared with respect to cardiopulmonary exercise performance following clinical recovery.

Due to the unpredictable timing of SARS-CoV-2 infection and the absence of routine pre-infection cardiopulmonary exercise testing (CPET) data for all participants, individual pre-infection baseline testing was not available. Therefore, the primary outcome of the study was post-infection cardiopulmonary functional capacity, assessed under standardized laboratory conditions before return to full training.

The study protocol was approved by the Research Ethics Committee of Singidunum University (protocol code 759-1; 4 June 2026) and conducted in accordance with the Declaration of Helsinki. Ethical approval covered the retrospective scientific analysis and publication of previously collected anonymized clinical data. All participants provided written informed consent after receiving detailed verbal and written explanations of the procedures and potential risks.

### 2.1. Participants

A total of 220 male elite professional team-sport athletes were included in the study, comprising 112 soccer players and 108 basketball players. All participants were members of professional clubs competing in the highest national-level leagues in Serbia. They underwent regular medical screening as part of their competitive and club-related medical obligations.

Most CPET assessments were performed during the competitive part of the season. All participants were professional team-sport athletes involved in regular club training and competition schedules. However, individual weekly training duration and load before infection were not systematically available from the medical records.

The analytical sample comprised athletes who underwent routine post-infection CPET and for whom complete clinical and CPET records were available. The sample was not prospectively recruited as a consecutive series of all athletes infected during the study period.

Participants were allocated to two groups based on SARS-CoV-2 vaccination status at the time of confirmed infection. The unvaccinated group included 101 athletes with PCR-confirmed SARS-CoV-2 infection who had not received a SARS-CoV-2 vaccine prior to infection. The vaccinated group included 119 athletes with PCR-confirmed SARS-CoV-2 infection after receiving two doses of a SARS-CoV-2 vaccine.

Vaccinated and unvaccinated athletes were assessed during partly overlapping portions of the study period. However, most unvaccinated athletes were assessed earlier, whereas most vaccinated athletes were assessed later.

Vaccination was voluntary, and athletes received one of the vaccines available in Serbia during the study period. Among the vaccinated athletes, 35 (29.4%) received the Pfizer-BioNTech vaccine, 53 (44.5%) received the Sinopharm vaccine, and 31 (26.1%) received the AstraZeneca vaccine. Vaccine type was not used as a grouping variable in the present analysis.

### 2.2. Inclusion and Exclusion Criteria

Inclusion criteria were male professional soccer or basketball player status, active membership in a professional club at the time of infection, at least six years of competitive experience, first laboratory-confirmed SARS-CoV-2 infection, documented vaccination status at the time of infection, completion of the required isolation period, clinical recovery, a negative PCR test before assessment, medical clearance for maximal cardiopulmonary exercise testing, and availability of complete CPET data obtained before return to full training.

Exclusion criteria were previous SARS-CoV-2 infection; musculoskeletal injury or illness requiring absence from training for more than four weeks during the preceding 12 months; known cardiovascular, pulmonary, or metabolic disease unrelated to SARS-CoV-2; persistent post-COVID-19 symptoms preventing maximal exercise testing; incomplete recovery; or failure to meet safety criteria for CPET.

### 2.3. Testing Procedure

**Figure 1:**
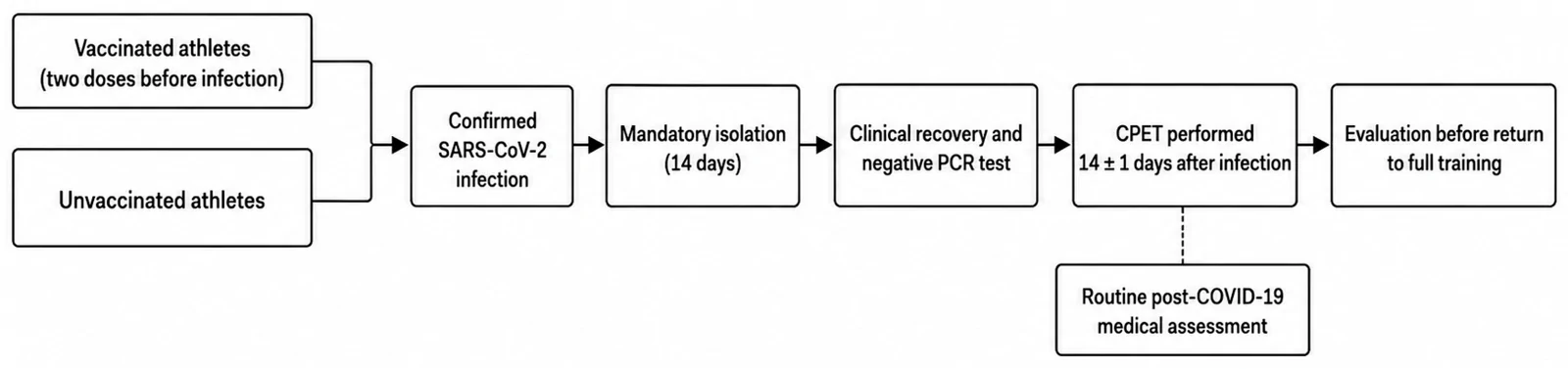
Schematic overview of the study design and timing of assessments.

Following laboratory confirmation of SARS-CoV-2 infection, all athletes completed the required isolation period and were temporarily restricted from full training. During the mandatory 14-day isolation period, the athletes did not participate in organized training. Cardiopulmonary exercise testing was performed 14 ± 1 days after confirmed SARS-CoV-2 infection, following clinical recovery and a negative PCR test (Figure 1).

Before testing, all participants underwent medical evaluation to confirm the absence of symptoms or contraindications to maximal exercise. This evaluation included assessment of persistent respiratory, cardiovascular, and general post-COVID-19 symptoms relevant to safe return-to-play and maximal exercise testing. The pre-CPET medical evaluation also included resting 12-lead electrocardiography and routine laboratory screening, including C-reactive protein, high-sensitivity cardiac troponin T, N-terminal pro-B-type natriuretic peptide, and D-dimer, as part of the clinical clearance process for maximal exercise testing. These assessments were used for clinical screening and were analyzed descriptively as pre-CPET screening characteristics rather than as primary or secondary study outcomes. CPET was performed before return to full training in order to assess early post-infection cardiopulmonary functional status under standardized laboratory conditions.

### 2.4. Cardiopulmonary Exercise Testing and Measured Variables

All participants underwent maximal cardiopulmonary exercise testing in an authorized sports medicine and exercise physiology laboratory. Testing was performed on a motorized treadmill (HP-COSMOS^®^, Nußdorf, Germany). Gas exchange was measured breath-by-breath using a calibrated metabolic cart (Quark CPET, COSMED^®^, Rome, Italy) with OMNIA software (version 2.2; COSMED, Rome, Italy). A 12-lead resting electrocardiogram (Fukuda^®^, Fukuoka, Japan) was recorded before testing. All tests were supervised by experienced sports medicine physicians.

The standardized treadmill protocol for professional athletes consisted of an initial speed of 6 km·h^−1^ at a constant incline of 3%. Running speed was increased by 1 km·h^−1^ every 40 s until volitional exhaustion or until maximal effort criteria were achieved, as previously described by Stojmenovic et al. [4].

The first ventilatory threshold (VT1) was determined using the V-slope method as the point at which carbon dioxide production began to increase disproportionately relative to oxygen uptake. This determination was supported by an increase in the ventilatory equivalent for oxygen without a simultaneous increase in the ventilatory equivalent for carbon dioxide. The second ventilatory threshold (VT2) was identified as the respiratory compensation point, characterized by a further nonlinear increase in minute ventilation, an increase in the ventilatory equivalents for both oxygen and carbon dioxide, and a decrease in end-tidal carbon dioxide pressure. Ventilatory thresholds were determined by visual inspection of breath-by-breath data by experienced sports medicine physicians [5].

A test was considered maximal if at least two of the following criteria were met: attainment of ≥90% of age-predicted maximal heart rate, calculated as 220 − age; a plateau in oxygen uptake despite increasing workload, defined as an increase of <150 mL·min^−1^; respiratory exchange ratio ≥1.20; or volitional exhaustion.

The following cardiopulmonary parameters were analyzed: maximal oxygen uptake (VO_2_max, mL·kg^−1^·min^−1^), oxygen uptake at the first ventilatory threshold (VO_2_@VT1, mL·kg^−1^·min^−1^), respiratory exchange ratio (RER), maximal minute ventilation (VEmax, L·min^−1^), ventilatory efficiency expressed as VE/VCO_2_, oxygen pulse (O_2_/HR, mL·beat^−1^), heart rate at the first and second ventilatory thresholds (HR@VT1 and HR@VT2), maximal heart rate (HRmax), and heart rate at 1, 2, and 3 min of recovery after exercise termination.

### 2.5. Statistical Analysis

No prospective sample-size calculation was performed because of the retrospective observational design. The final sample size was determined by the number of available records that met the predefined eligibility criteria and contained complete CPET data. Descriptive statistics were calculated for all variables. Continuous variables are presented as means ± standard deviations, while categorical variables are presented as frequencies and percentages.

Distributional characteristics of continuous variables were evaluated using histogram inspection, Q–Q plots, and the Kolmogorov–Smirnov test. Homogeneity of variance was assessed using Levene’s test.

For each cardiopulmonary exercise testing variable, a two-way between-subject analysis of variance was performed, with vaccination status (unvaccinated vs. vaccinated) and sport (basketball vs. soccer) entered as fixed factors. The main effects of vaccination status and sport, as well as the vaccination status × sport interaction, were examined. When the interaction effect was not statistically significant, the main effect of vaccination status was interpreted as the overall between-group difference adjusted for sport. Sport-adjusted between-group mean differences and corresponding 95% confidence intervals were estimated from the estimated marginal means and expressed as vaccinated minus unvaccinated athletes. When a significant interaction was observed, simple main effects using Bonferroni-adjusted pairwise comparisons were examined to compare vaccinated and unvaccinated athletes within each sport subgroup.

Maximal oxygen uptake (VO_2_max) was considered the primary outcome, while the remaining cardiopulmonary exercise testing variables were treated as secondary outcomes. Analyses of secondary outcomes were considered exploratory, and these findings were interpreted as supportive rather than confirmatory. Because multiple secondary outcomes were examined, the possibility of type I error was considered when interpreting isolated statistically significant findings. Effect sizes for ANOVA effects were expressed as partial eta squared (ηp^2^) and interpreted using conventional thresholds: small (≥0.01), medium (≥0.06), and large (≥0.14) [19].

Categorical variables, including sport distribution according to vaccination status, were compared using the chi-square test. Statistical significance was set at *p* < 0.05. All analyses were conducted using SPSS software, version 20.0 (IBM Corp., Armonk, NY, USA).

## 3. Results

All participants successfully completed the cardiopulmonary exercise testing protocol and met the criteria for maximal effort. A total of 220 male elite professional team-sport athletes were included in the analysis, comprising 101 unvaccinated and 119 vaccinated athletes.

Participant characteristics according to vaccination status are presented in Table 1. The vaccinated and unvaccinated groups did not differ significantly in age (*p* = 0.385), body height (*p* = 0.467), body mass (*p* = 0.404), BMI (*p* = 0.556), or sport distribution (*p* = 0.701). No significant anthropometric differences between vaccination groups were observed within the basketball or soccer subgroups.

Pre-CPET inflammatory and cardiac biomarker values according to vaccination status are presented in Table 2. Mean CRP, D-dimer, NT-proBNP, and hs-cTnT values were within the corresponding laboratory reference ranges in both groups.

Cardiopulmonary exercise testing parameters according to vaccination status are presented in Table 3. A two-way ANOVA was performed for each CPET variable, with vaccination status and sport entered as fixed factors. After accounting for sport, a significant main effect of vaccination status was observed for VO_2_max, VO_2_@VT1, VE/VCO_2_, oxygen pulse, HR@VT1, and HR@VT2. Vaccinated athletes demonstrated higher VO_2_max, higher VO_2_@VT1, higher oxygen pulse, and higher heart rate at both ventilatory thresholds, as well as lower VE/VCO_2_ values, compared with unvaccinated athletes. After adjustment for sport, vaccinated athletes had a 2.47 mL·kg^−1^·min^−1^ higher VO_2_max than unvaccinated athletes (adjusted mean difference: 2.47; 95% CI: 1.30 to 3.63).

A significant main effect of vaccination status was also observed for RER; however, this difference was small and was interpreted as a secondary effort-related parameter rather than as a central indicator of post-infection cardiopulmonary functional performance.

**Figure 2:**
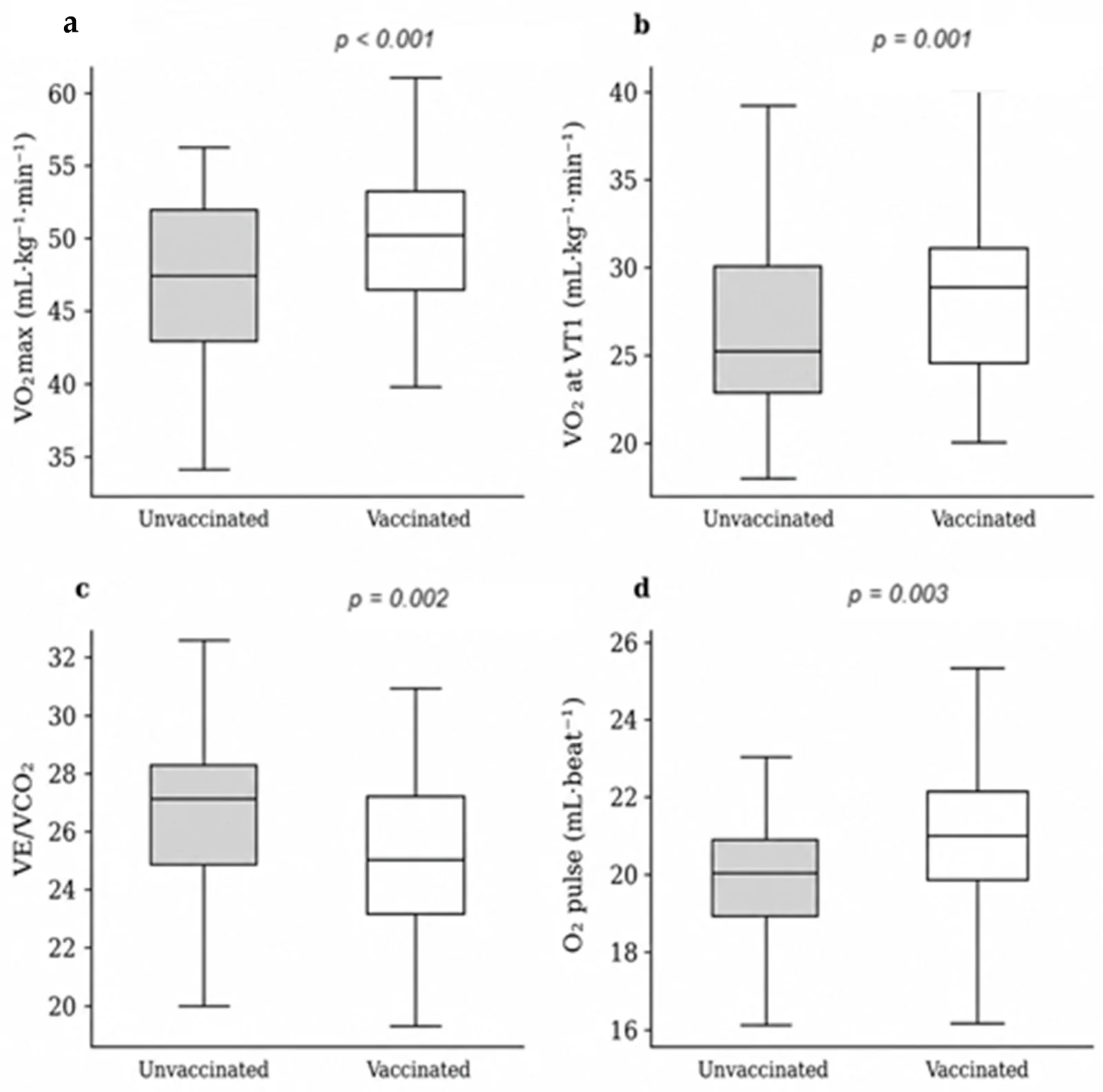
Key post-infection cardiopulmonary exercise testing parameters according to vaccination status. Boxplots show maximal oxygen uptake (VO_2_max) (**a**), oxygen uptake at the first ventilatory threshold (VO_2_@VT1) (**b**), ventilatory efficiency (VE/VCO_2_) (**c**), and oxygen pulse (**d**) in unvaccinated and vaccinated athletes after SARS-CoV-2 infection. Reported *p*-values refer to the main effect of vaccination status from two-way ANOVA with vaccination status and sport entered as fixed factors.

The main differences in key cardiopulmonary exercise testing parameters are illustrated in Figure 2. Vaccinated athletes demonstrated higher VO_2_max, higher VO_2_@VT1, and higher oxygen pulse, as well as lower VE/VCO_2_ values, compared with unvaccinated athletes.

Sport-specific descriptive values were examined to determine whether the direction of differences between vaccinated and unvaccinated athletes was similar in basketball and soccer players (Table 4). For the selected CPET outcomes, vaccinated athletes showed higher VO_2_max, higher VO_2_@VT1, higher oxygen pulse, and lower VE/VCO_2_ values in both sport subgroups. The vaccination status × sport interaction was not significant for any of these parameters, indicating that the association between vaccination status and the main CPET outcomes did not differ significantly between basketball and soccer players.

## 4. Discussion

The present study compared post-infection cardiopulmonary exercise performance between vaccinated and unvaccinated elite male team-sport athletes following confirmed SARS-CoV-2 infection. The main finding was that vaccination status was associated with differences in several post-infection CPET parameters, particularly higher VO_2_max, higher VO_2_@VT1, higher oxygen pulse, and lower VE/VCO_2_ values. These differences remained evident after accounting for sport, and no significant vaccination status × sport interaction was observed for the main CPET outcomes, suggesting that the association between vaccination status and post-infection functional performance was generally consistent across basketball and soccer players. With the exception of VO_2_max, which was defined as the primary outcome, the remaining findings should be interpreted as exploratory and supportive.

The higher VO_2_max observed in vaccinated athletes was consistent with a more favorable maximal aerobic profile during the early post-infection period. VO_2_max is one of the most important integrative indicators of oxygen transport and utilization, and even relatively small reductions may be relevant in elite athletes, particularly in intermittent team sports that require repeated high-intensity efforts. Previous studies have reported that SARS-CoV-2 infection may negatively affect aerobic capacity and cardiopulmonary function in athletes, including professional soccer players, professional basketball players, and unvaccinated athletic populations [4,14,15,16]. In this context, the higher VO_2_max values observed in vaccinated athletes suggest that prior vaccination status may be associated with a more favorable post-infection aerobic profile. Similarly, VO_2_@VT1 was significantly higher in vaccinated athletes, suggesting a more favorable submaximal aerobic profile. This parameter is particularly relevant for soccer and basketball players, as the ability to sustain exercise below or around the first ventilatory threshold contributes to repeated efforts, recovery between high-intensity actions, and overall match demands [14,17].

Ventilatory efficiency, expressed as VE/VCO_2_, was also more favorable in vaccinated athletes. Lower VE/VCO_2_ values indicate more efficient ventilation relative to carbon dioxide production and are commonly interpreted as a marker of better integrated pulmonary and cardiopulmonary function during exercise [18]. This parameter may be particularly sensitive to post-COVID-19 alterations in ventilatory control, pulmonary perfusion, and ventilation–perfusion matching [4,6]. In the present study, vaccinated athletes demonstrated lower VE/VCO_2_ values than unvaccinated athletes, suggesting a more favorable ventilatory response during maximal exercise testing. Together with the higher VO_2_max and VO_2_@VT1 values, this finding was consistent with a more favorable post-infection cardiopulmonary profile in vaccinated athletes.

Oxygen pulse was significantly higher in vaccinated athletes compared with unvaccinated athletes. Since oxygen pulse reflects the amount of oxygen consumed per heartbeat, it is commonly used as an indirect indicator of stroke volume and peripheral oxygen extraction during exercise [18,20]. Lower oxygen pulse values after SARS-CoV-2 infection may indicate transient impairments in cardiovascular function, oxygen delivery, or peripheral oxygen utilization [4]. Although previous studies examining vaccination and exercise responses have generally reported minimal adverse effects of vaccination on cardiopulmonary performance [8,21], the present findings suggest an association between prior vaccination status and a more favorable oxygen-pulse response during post-infection CPET.

Heart rate responses provide additional information on exercise tolerance and physiological effort during CPET. In the present study, vaccinated athletes demonstrated higher HR@VT1 and HR@VT2, while HRmax did not differ significantly between groups. These findings suggest that vaccinated athletes reached ventilatory thresholds at higher heart rates, which should be interpreted together with their higher VO_2_@VT1 values rather than as an isolated indicator of superior chronotropic function. The absence of between-group differences in HRmax is consistent with previous evidence indicating minimal effects of COVID-19 vaccination on maximal chronotropic responses during graded exercise testing [8,21]. Similarly, heart rate values during the first three minutes of recovery did not differ significantly between groups, suggesting no clear difference in early post-exercise heart rate response. A small significant difference was observed for RER; however, because RER primarily reflects exercise effort and substrate utilization at peak intensity and because both groups achieved values consistent with maximal or near-maximal effort, this finding is unlikely to represent a clinically meaningful difference in functional capacity [18,22].

An important finding of the present study was that the association between vaccination status and the main CPET outcomes did not differ significantly between basketball and soccer players. Although these sports differ in movement patterns, match demands, and typical physiological profiles, vaccinated athletes showed a similar direction of differences in both sport subgroups, with higher VO_2_max, higher VO_2_@VT1, higher oxygen pulse, and lower VE/VCO_2_ values. The absence of significant vaccination status × sport interactions for these parameters suggests that the observed association between prior vaccination and more favorable post-infection cardiopulmonary exercise performance was not limited to one specific team sport. This supports the relevance of the findings for elite male team-sport athletes more broadly, while still acknowledging that sport-specific physiological characteristics should be considered when interpreting CPET outcomes [14,15,16].

The normal inflammatory and cardiac biomarker findings in both groups reduce the likelihood that overt ongoing systemic inflammation or myocardial injury accounted for the observed CPET differences. Nevertheless, these screening results do not replace information on individual pre-infection fitness, habitual training volume, or detailed acute symptom burden. Importantly, CPET was performed during the early post-infection period, and athletes with persistent symptoms were excluded. Therefore, the present findings do not provide information on Long COVID or persistent post-COVID-19 functional impairment.

The present findings should be interpreted in light of several limitations. First, individual pre-infection CPET data were not available; therefore, the results represent between-group differences in early post-infection functional status rather than direct longitudinal changes from pre-infection baseline. In addition, the study included only athletes who had clinically recovered, received medical clearance, and were able to complete maximal CPET during the defined post-infection assessment period. Athletes with delayed recovery, persistent symptoms, or contraindications to maximal testing were therefore not represented, which may have introduced selection bias and limited the generalizability of the findings to all athletes with SARS-CoV-2 infection. However, all athletes refrained from training during the mandatory isolation period and underwent CPET before returning to full training, which reduced variability in recent training exposure immediately before testing. Furthermore, all participants were male elite professional team-sport athletes, competed at comparable levels, had at least six years of competitive experience, and underwent regular medical screening, which reduces the likelihood of major baseline differences between groups. Moreover, sport-specific performance outcomes, including training performance, match performance, and time to full competitive return, were not available. Therefore, the observed differences in CPET parameters cannot be directly translated into differences in actual athletic or competitive performance. In addition, multiple secondary CPET outcomes were examined without formal adjustment for multiplicity; therefore, these findings should be interpreted as exploratory and require confirmation in future studies. Second, vaccination status was closely associated with calendar period. Although limited temporal overlap existed between the two groups, most unvaccinated athletes were assessed earlier and most vaccinated athletes later. Therefore, residual confounding related to temporal changes in clinical management, return-to-play procedures, and other pandemic-related circumstances cannot be excluded [4,5,6]. In addition, residual confounding related to habitual training volume, acute infection characteristics, and symptom duration cannot be excluded because these variables were not consistently available for analysis. Third, vaccine type and the time interval between vaccination and infection were not controlled. Although vaccine distribution was available descriptively, the study was not designed to compare individual vaccine platforms, and such comparisons would have been difficult to interpret because vaccine availability, timing of vaccination, and pandemic period were closely interrelated. Finally, the analysis was limited to male soccer and basketball players, and the findings should not be generalized to female athletes, individual-sport athletes, or non-elite populations without further investigation.

Despite these limitations, the present study provides clinically relevant information for sports medicine and return-to-play practice. The observed pattern of more favorable CPET parameters in vaccinated athletes is consistent with previous evidence reporting associations between vaccination and reduced disease severity, training disruption, and adverse functional consequences in athletic and physically active populations [9,12,13]. Future studies should use longitudinal designs with repeated pre- and post-infection CPET assessments, examine the effects of booster vaccination and vaccine type, and consider variant-specific outcomes.

## 5. Conclusions

Prior SARS-CoV-2 vaccination was associated with a more favorable early post-infection cardiopulmonary exercise profile in elite male team-sport athletes. Vaccinated athletes demonstrated higher VO_2_max, higher VO_2_@VT1, higher oxygen pulse, and lower VE/VCO_2_ values compared with unvaccinated athletes, after accounting for sport. The absence of significant vaccination status × sport interactions for the main CPET outcomes suggests that this pattern was generally consistent across soccer and basketball players. These findings suggest that vaccination status may be considered as one of several contextual factors when interpreting early post-infection CPET findings in professional athletes. However, due to the observational design and the absence of individual pre-infection CPET data, the findings should be interpreted as associations rather than evidence of a direct causal effect.

## Figures and Tables

**Table 1:** Participant characteristics according to vaccination status.

Variable	Unvaccinated Athletes	Vaccinated Athletes	*p*-Value
Study sample size (%)	101 (45.9)	119 (54.1)	
Age (M ± SD)	23.35 ± 4.58	22.80 ± 4.71	0.385
Body height (cm)	190.70 ± 11.59	189.58 ± 11.20	0.467
Body mass (kg)	86.70 ± 14.08	85.13 ± 13.78	0.404
BMI (kg·m^−2^)	23.67 ± 1.59	23.54 ± 1.76	0.556
Basketball			
Sample size (%)	51 (50.5)	57 (47.9)	
Age (M ± SD)	23.71 ± 4.95	24.12 ± 5.17	0.670
Body height (cm)	200.20 ± 7.18	198.39 ± 9.06	0.256
Body mass (kg)	98.09 ± 9.29	96.40 ± 10.49	0.380
BMI (kg·m^−2^)	24.44 ± 1.48	24.46 ± 1.61	0.949
Soccer			
Sample size (%)	50 (49.5)	62 (52.1)	
Age (M ± SD)	22.98 ± 4.18	21.58 ± 3.91	0.071
Body height (cm)	181.02 ± 5.68	181.48 ± 5.33	0.658
Body mass (kg)	75.09 ± 6.62	74.76 ± 6.18	0.790
BMI (kg·m^−2^)	22.89 ± 1.30	22.69 ± 1.46	0.454

Values are presented as mean ± standard deviation or n (%). Between-group differences in age, body height, body mass, and BMI were analyzed using independent-sample *t*-tests.

**Table 2:** Pre-CPET inflammatory and cardiac biomarkers according to vaccination status.

Variable	Unvaccinated Athletes	Vaccinated Athletes	Reference Range
CRP (mg/L)	2.70 ± 1.13	2.91 ± 0.92	<10
D-dimer (mg/L)	0.18 ± 0.09	0.23 ± 0.08	<0.50
NT-proBNP (pg/mL)	16.39 ± 5.63	15.87 ± 5.80	<125
hs-cTnT (ng/L)	3.97 ± 1.45	4.52 ± 1.65	<14

Values are presented as mean ± standard deviation. CRP, C-reactive protein; NT-proBNP, N-terminal pro-B-type natriuretic peptide; hs-cTnT, high-sensitivity cardiac troponin T. Biomarker measurements were used as part of the clinical screening before maximal CPET.

**Table 3:** CPET parameters according to vaccination status: main effect from two-way ANOVA.

Variable	Unvaccinated Athletes	Vaccinated Athletes	Adjusted Mean Difference (95% CI)	F (1216)	*p*-Value	ηp^2^
VO_2_max (mL·kg^−1^·min^−1^)	47.22 ± 4.92	49.76 ± 4.47	2.47 (1.30 to 3.63)	17.46	<0.001	0.075
VO_2_@VT1 (mL·kg^−1^·min^−1^)	25.92 ± 4.57	28.04 ± 4.97	2.15 (0.87 to 3.42)	11.00	0.001	0.048
VE/VCO_2_	26.45 ± 2.70	25.19 ± 3.09	−1.26 (−2.04 to −0.48)	10.11	0.002	0.045
RER	1.18 ± 0.06	1.17 ± 0.04	−0.016 (−0.029 to −0.003)	6.08	0.014	0.027
O_2_ pulse (mL·beat^−1^)	20.05 ± 1.90	20.88 ± 2.18	0.83 (0.27 to 1.38)	8.72	0.003	0.039
VEmax (L·min^−1^)	143.50 ± 28.14	141.59 ± 20.26	−1.79 (−8.25 to 4.66)	0.30	0.585	0.001
HR@VT1 (beats·min^−1^)	142.04 ± 9.40	145.46 ± 10.08	3.27 (0.78 to 5.76)	6.72	0.010	0.030
HR@VT2 (beats·min^−1^)	167.49 ± 10.93	171.22 ± 9.45	3.55 (1.00 to 6.10)	7.53	0.007	0.034
HRmax (beats·min^−1^)	186.83 ± 8.78	185.87 ± 8.60	−1.08 (−3.34 to 1.18)	0.89	0.347	0.004
HR at 1 min recovery (beats·min^−1^)	162.27 ± 12.07	159.79 ± 13.39	−2.67 (−5.98 to 0.64)	2.53	0.113	0.012
HR at 2 min recovery (beats·min^−1^)	135.08 ± 15.57	131.38 ± 15.72	−3.89 (−7.99 to 0.20)	3.52	0.062	0.016
HR at 3 min recovery (beats·min^−1^)	122.36 ± 14.69	120.03 ± 14.44	−2.45 (−6.31 to 1.41)	1.57	0.212	0.007

Values are presented as unadjusted means ± standard deviations. F- and *p*-values refer to the main effect of vaccination status from the two-way between-subject ANOVA, with vaccination status and sport entered as fixed factors; ηp^2^ = partial eta squared; adjusted mean differences are expressed as vaccinated minus unvaccinated athletes and are based on estimated marginal means from the two-way ANOVA adjusted for sport.

**Table 4:** Sport-specific descriptive values and vaccination status × sport interaction for selected CPET parameters.

Variable	Sport	Unvaccinated Athletes	Vaccinated Athletes	F (1216)	*p*-Value	ηp^2^
VO_2_max (mL·kg^−1^·min^−1^)	Basketball	45.05 ± 4.72	48.44 ± 4.77	2.43	0.120	0.011
	Soccer	49.43 ± 4.10	50.98 ± 3.83			
VO_2_@VT1 (mL·kg^−1^·min^−1^)	Basketball	26.68 ± 4.99	28.35 ± 4.70	0.55	0.458	0.003
	Soccer	25.14 ± 4.00	27.76 ± 5.24			
VE/VCO_2_	Basketball	26.30 ± 2.80	25.11 ± 2.36	0.03	0.875	<0.001
	Soccer	26.60 ± 2.60	25.27 ± 3.65			
O_2_ pulse (mL·beat^−1^)	Basketball	20.06 ± 2.31	20.84 ± 2.14	0.03	0.870	<0.001
	Soccer	20.04 ± 1.39	20.92 ± 2.23			

Values are presented as mean ± standard deviation; F- and *p*-values refer to the vaccination status × sport interaction from the two-way between-subject ANOVA; ηp^2^ = partial eta squared.

## Data Availability

The datasets presented in this article are not readily available because the data are part of an ongoing study. Requests to access the datasets should be directed to the corresponding authors.
